# Using Exploratory Focus Groups to Inform the Development of Targeted COPD Self-Management Education DVDs for Rural Patients

**DOI:** 10.1155/2010/450418

**Published:** 2010-06-29

**Authors:** Michael Stellefson, Beth H. Chaney, J. Don Chaney

**Affiliations:** ^1^Department of Health Education & Behavior, University of Florida, P.O. Box 261954, FLG 23, Gainesville, FL 32611, USA; ^2^College of Health and Human Performance, University of Florida, 242B Florida Gym, P.O. Box 118200, Gainesville, FL 32611, USA

## Abstract

This exploratory study assessed the self-management learning needs, experiences, and perspectives of COPD patients treated at a Certified Federal Rural Health Clinic to inform the development of a COPD self-management DVD. A purposive, homogeneous sample of COPD patients participated in focus group interviews. Data from these interviews were referenced to edit a library of Rvision COPD self-management DVDs into a single condensed DVD containing only the most pertinent self-management topics. Patients reported a lack of knowledge and skill development related to purse lipped breathing, controlled coughing, and stress management; while medication management skills were found to be quite adequate. Engaging rural communities in formal qualitative inquiries to describe COPD specific needs for self-management may lead to future use of educational technologies aimed at improving quality of life for these rural, hard to reach populations.

## 1. Introduction

Chronic obstructive pulmonary disease (COPD) is a preventable and treatable disease, characterized by progressive airflow limitation that is not fully reversible and is associated with an abnormal inflammatory response of the lung to noxious particles or gases [1]. Airflow limitation is signified by two specific respiratory diseases, emphysema and/or chronic bronchitis. Dyspnea, or shortness of breath, is the hallmark symptom of COPD and is a major cause of disability and anxiety associated with the disease. The prevalence, morbidity, and mortality associated with COPD are generally directly related to the prevalence of tobacco smoking and increased age [2]. COPD is the fourth leading cause of death in the United States (following heart disease, cancer, and stroke) and is the only major cause of death in America for which no significant decrease in morbidity or mortality has been observed over the past 20 years [3, 4]. In effect, by the year 2020, COPD is estimated to be the 3rd leading cause of death and 5th leading cause of disability in the world [5, 6].

COPD patients are faced with multiple health responsibilities, such as preventing and managing shortness of breath and handling prescribed medications. It has been suggested that healthcare professionals focus attention on the care processes necessary for patients to cope with complications that arise due to this debilitating disease [7]. *Disease management* is an approach which coordinates resources across the health care system with the aim of increased patient knowledge and control over disease [8]. For COPD patients, pulmonary rehabilitation is one such disease management option. These programs are usually hospital run and include supervised regimens of physical exercise, behavioral modification, nutrition counseling, and disease education. The first enrollment in pulmonary rehab is normally covered by Medicare for patients who qualify; however, patients who wish to continue rehabilitation after the first round of therapy must usually pay for it out of pocket. This type of maintenance program can prove to be quite costly for patients, especially those with limited monetary resources. Therefore, attempts at COPD *self-management* in the home environment, with no requirements for specialized facilities or group meetings, are ideal.

COPD self-management refers to engaging in activities that promote adequate inhalation technique, building physiologic reserves, preventing adverse health outcomes, complying with recommended treatment protocols, monitoring respiratory and emotional status and making appropriate management decisions on the basis of this self-monitoring, and managing the effects of illness on self-esteem and coping skills [9]. Chronic disease self-management programs promote active patient participation and focus on acquisition and implementation of learned skills and enhancing patient self-confidence related to disease management decision making [10]. In systematic reviews of the efficacy of COPD self-management education programs, an association was established between self-management education and improved health-related quality of life (HRQoL), with no indication of detrimental effects on other related health outcomes [11]. In light of the effectiveness of some COPD self-management programs, health educators have been encouraged to develop cost-effective, readily accessible interventions which (a) define the true effective educational elements of COPD self-management and (b) facilitate the acquisition of self-management skills and behavior change. Previous work indicates that adults use a variety of strategies to adapt to chronic disability [12], each of which may impact health and health-related quality of life (HRQoL) in unique and disparate ways. Moreover, understanding self-management behaviors is complex and represents a “black box” for health care professionals [13].

It is known, however, that targeted health education plays a vital role in improving skills, coping ability and HRQoL among patients with COPD [14–16]. Targeted patient education may not improve exercise tolerance or lung function, but it can have a dramatic impact on patients' ability to deal with the disability caused by COPD. Rabe and colleagues advise that, “Education should be…directed at improving quality of life, simple to follow, practical and appropriate to the intellectual and social skills of the patient” [1, page 541], given that patients differ in terms of the depth and type of information that they seek [17]. Moreover, patient-centered education developed in accordance with specific needs is a key factor in managing COPD [18, 19]. Information needs assessments attempt to ascertain the opinions of learners regarding what information *they* want to learn, what educational topics *they* believe are important and which self-management topics *need* to be addressed [20].

Given time constraints, the majority of primary care physicians are unable to provide sufficient “in-person” education to patients regarding COPD self-management. In addition, some physicians do not feel prepared to teach patients within the clinical environment due to the low priority that patient education is given by managed care administrators. The absence of third-party reimbursement to support patient education makes teaching and learning between provider and patient quite difficult. This is problematic, because patients report not having their disease-related concerns addressed during routine consultations [21]. Patients suffering from COPD report widespread dissatisfaction with the self-management education they are provided [22, 23]. Because of this, some COPD patients do not feel connected to their health care provider, which affects patient decision making when attempting to manage disease [24]. COPD patients prefer to be involved in their own medical decision making and respond better if they are included in the process [25, 26].

## 2. Literature Review

Undoubtedly, there seems to be a paucity of patient-centered interventions that take into account differing patient perspectives and knowledge of COPD self-management by way of a needs assessment [27]. This is disappointing, given that patient educators are taught to recognize dissimilar concerns brought to light across divergent populations [28] and develop programs in accordance with a mutually understood care plan [10]. Qualitative inquiry can assist practitioners in developing programs which address self-management issues valued by patients. Focus group interviews have proven to be a useful research technique for identifying older adults' beliefs and needs regarding specific health topics. Cicutto et al. [13] conducted focus groups with a purposive sample of patients suffering from COPD in order to understand the self-management activities of COPD patients and the meaning that patients associate with these activities. Constant comparative analysis was used to examine focus group transcripts. The major theme which emanated from these focus group sessions centered on the idea of “surviving COPD,” which ultimately included adjusting physically and emotionally to COPD in order to achieve a satisfactory HRQoL. A common adjustment adopted by patients was a shift towards more planning, pacing, and prioritizing for activities of daily living and disease management [13]. Planned, energy-saving activities included breathing exercises, slow walks, and more sedentary forms of recreation. Breathing exercises, along with medication management, were identified as useful strategies for enabling activities of daily living. Aerobic and strengthening exercises were identified as dispensable due to lack of motivation and unpleasant feelings following physical exertion. One limitation of this study was the lack of representation among racial and ethnic minorities, whom may have separate and distinct perspectives of COPD self-management.

In order to understand statistically nonsignificant results of a quantitative investigation measuring change in HRQoL after COPD self-management intervention [29], Monninkhof et al. [30] conducted in-depth interviews with a purposive sample of 20 participants. Patients reported that self-management education helped them distribute energy evenly, control symptoms more effectively, and manage medication intake. Increased confidence coping with disease symptoms was also reported, which corroborated findings by Camp et al. [31], who characterized patient confidence over chronic disease as an important mediating variable along the continuum from self-management skill adoption to HRQoL improvement.

## 3. Rationale and Theoretical Framework

Older adults suffering from COPD have unique preferences for the manner in which they obtain self-management education [32, 33]. It has been suggested that older COPD patients appreciate and learn well from home-based educational programs that offer audiovisual media to transmit targeted self-management instruction [34–38]. In light of this, type III translational research should focus on disseminating innovative educational material through readily available technology in order to provide underserved COPD patients with customized resources. The aim of the present study was to assess the self-management learning needs, experiences and perspectives of COPD patients who were treated at a Certified Federal Rural Health Clinic in order to inform the development of a COPD self-management DVD.

There were two research questions developed to achieve the primary aim of the study: (1) what are the self-management experiences of patients with COPD who live in a rural setting? and (2) how do patients perceive the effects that self-management can have on their HRQoL?

Mass communication technologies can be utilized to provide patients with self-management skill development products which can help enhance self-management self-efficacy beliefs. Theories of *consumer information processing* (CIPT) provide a framework for understanding why people do or do not pay attention to, understand, and/or make use of consumer health information [39]. CIPT postulates that information must not only be available but also be wanted and believed useful by the consumer. Further, the consumer must possess the time and level of comprehension necessary to process the information presented, as consumer decision making involves multiple stages of acquiring, processing, learning, using and evaluating information [39, 40]. A central premise of CIPT is that individuals can process only a limited amount of information at one time [40]; thus, the information must be presented in such a way that the patient is able to comprehend the information that is provided.

## 4. Methods

Focus groups were conducted in hopes of achieving data saturation (i.e., no longer hearing new information), which would provide understanding as to the meaning that COPD patients associate with COPD self-management. Qualitative data yield thick and rich descriptions which are contextualized based on the multicausal experiences people endure [41]; thus, this type of inquiry makes sense of experiences by uncovering the meanings that people associate with various events [42]. 

### 4.1. Setting

COPD patients were recruited from a Certified Federal Rural Health clinic located in Butler, Alabama. Butler is a rural town located in Choctaw County, a low socioeconomic district. In 2003, it was reported that almost 20% of the population in Butler lived below the poverty line [43]. Twenty-four percent of Choctaw County is farmland, and Choctaw is one of 42 counties classified as “rural” in Alabama [44], with rural being defined as population living in towns outside a commuting zone of larger urban areas. Butler is one such diverse, rural municipality, with 43.9% of its citizens being of African-American decent, and 52.8% being female. In addition, over 15% of Butler's population are comprised of citizens over 65 [43]. Choctaw County has been designated as a Health Professional Shortage Area by the Federal Office of Health Professions [45].

### 4.2. Sample

A purposive, homogeneous sample of COPD patients was recruited within Choctaw County using various proactive recruitment strategies such as physician referrals, newspaper advertisements, and telephone contacts. Homogeneous sampling was utilized, because participants needed to possess a clinical diagnosis of COPD. This sampling approach is often used to select focus groups [46]. Within each of two focus groups, 6 patients were recruited. This number was selected for each group based on three reasons: (a) best practice minimum recommendations for focus group research [47, 48] and (b) the desire to obtain an in-depth understanding of the topic and high involvement from the participants [47]. The inclusion criteria for participation were: (a) adults 50 years of age or older, (b) clinical diagnosis of COPD (i.e., chronic bronchitis or emphysema), (c) presence of dyspnea, and (d) provision of informed consent. The exclusion criterion for participation was past participation in structured pulmonary rehabilitation programs wherein self-management activities were pre-selected.

### 4.3. Procedures

Preliminary information, such as patient medical diagnosis and age, was obtained through a telephone interview with potential patients. Interested patients were screened for gender and race/ethnicity to ensure comparable numbers of men and women and desired diversity of race. In addition, patients were asked to report their age, marital/relationship status, educational level, income and living arrangements. Informed consent was obtained from each participant before their participation. Each participant was provided with refreshments during the one-hour focus group along with a $20 honorarium for their participation. Institutional Review Board (IRB) approval for this study was secured. At the end of each focus group session, member checking was done to confirm the initial interpretations of the mediator and to enhance the descriptive and interpretive validity of the data analysis [49]. Focus group interviews were transcribed using paper and pencil with the aid of a digital audio recorder. Appendices A and B describe the rubric used by the moderator to conduct the interviews.

### 4.4. Data Analysis

Focus group data was analyzed using three distinct qualitative analysis tools: (a) method of deductive constant comparison; (b) classical content analysis, and (c) word count. Three tools were used to analyze the same data at the request of Leech and Onwuegbuzie [50], who state that using more than one type of analysis can augment the rigor and trustworthiness of qualitative findings through methodological triangulation. Constant comparison analysis [51] or “coding” was undertaken deductively to identify general, underlying themes within the focus group data. The entire data set acquired during the present study was read over and chunks of data (i.e., related portions of the transcript) were grouped into meaningful parts. Following this, the chunks were assigned to inductive codes which emerged. To determine the frequency of themes identified within the data, a classical content analysis procedure was employed to further augment the findings from the method of constant comparison [50]. Instead of creating themes from emergent codes, the number of times each code emerged was quantified for saturation across the focus group interviews. This was done to understand which codes were used most, thus cultivating a greater understanding of the most important concepts reported by the interviewees. If a greater number of codes were identified within a certain thematic category, then that category was deemed to be more relevant. Lastly, a word count method was used to quantify the number of times a specific word was used during the focus group discussions. Word count is especially useful for focus group analysis, because counts can help identify words spoken the most and those spoken the least. More important and noteworthy words were hypothesized to be used more often [52], thus providing more meaning from the conversation descriptions [53]. Additionally, word count assisted the researcher in identifying patterns, verifying hypotheses and maintaining analytic integrity and rigor [54].

## 5. Results

Each of the focus groups (*n* = 2) was held in a group meeting room. Table 1 describes the demographic characteristics of the 12 focus group participants. Over half of the participants were female, and the majority did not finish high school. All participants reported an annual income between $15,000 and $24,999; thus, participants were of low socioeconomic status. Half of the focus group participants were married, yet an almost equal proportion were widowed (33.33%) or single (16.67%). The overwhelming majority of participants (83.33%) lived with family, and the mean number of years participants reported being diagnosed with COPD was variable (mean = 6 years; SD = 4.43 years). Patients included in each of these focus groups represented both Caucasian and African American races. 

### 5.1. Constant Comparison Analysis

The three specific subthemes identified were (a) *adjusting physically to COPD*, (b) *using COPD self-management skills to cope physically*, and (c) *coming to terms with COPD and the lifestyle*. Table 2 summarizes the overall theme and the associated subthemes present within the focus group data.

#### 5.1.1. Surviving and Living Well with COPD

Almost all focus group participants spoke about the inherent difficulties living life while trying to manage symptoms caused by COPD. A variety of health problems were discussed, particularly with regards to patient difficulty balancing their lives with disease management. Two participants explicitly reported symptoms such as congestion, shortness of breath, difficulty coughing, sinus trouble, and wheezing. The majority of patients substantiated these symptom claims as being common. Patients indicated difficulty identifying coping strategies which could ameliorate these specific symptoms.

#### 5.1.2. Adjusting Physically to COPD

Physical adjustments to symptoms caused by COPD were reported by almost all members of the focus groups. Specifically, patients discussed their need to reduce the amount of movement that they engaged in from day to day. These problematic movements included basic activities of daily living such as: walking around the house, up and down stairs, and outside to take out the garbage. Participants also reported difficulty simply bending over and standing up straight without losing their breath. Because of these basic physical limitations, participants reported drastically reducing their overall activity levels:

“Everything I do I am out of breath and out of air. I find that it's sometimes worse than other times. I feel as if I have poor blood circulation to my legs as well, which makes me less wanting to walk around too much” (F2 P2 #1).

“Walking? I can't really walk too far at all now” (F1 P2 #3).

“If I am doing something, anything, and I seem to get real short of breath, then I just cut back. In fact, if you hold your breath a couple seconds longer than you should, then you have to try and catch your breath again” (F2 P1 #2).

“Every time I straighten up, I feel out of breath, even after I do something simple” (F2 P1 #9; F2 P1 #2; F2 P2 #1).

“Simply put, I am disabled” (F2 P1 #1).

In addition, female members of the focus groups talked about their difficulty performing daily cleaning chores around the house:

“Work is a lot harder for me now, specifically cooking and cleaning. I just can't cook and clean the way that I used to because when I try to do so, I lose my breath” (F1 P3 #2).

“For me, being a woman, I am finding that it is hard to sweep the floor. I have problems getting enough air in me when I'm doing it. When I bend down and dust to take care of the house, and I walk from room to room, and it just is so hard” (F2 P2 #1).

“The dust in the house and animal hair gives me problems as well. Using the Pine Sol and bleach when I am cleaning sometimes gives me an attack. The Lysol spray makes me cough” (F2 P1 #2).

Because of the difficulty patients reported accomplishing their activities of daily living, some noted the need to purposively plan how they went about moving. Patients noted the need to think about what they were going to do before they actually did it. In addition, they also felt the need to act in a delayed, deliberate manner to ensure that their actions did not result in shortness of breath. When patients neglected to engage in this thoughtful process, they inadvertently accelerated their movements, and brought on shortness of breath which was unanticipated and prolonged. These exacerbations occurred when patients attempted to do the most fundamental of tasks.

#### 5.1.3. COPD Self-Management: Adjusting Physically

Patients reported a widespread interest in wanting to learn more about COPD self-management strategies which could improve physical symptoms. While some patients reported engaging in some activities to self-manage COPD, others reported not knowing anything about what to do in terms of disease self-management. This was primarily because, for these patients, COPD was a newly encountered disease. For example, two patients expressed concerns about being unsure as to what could be done to improve the physical symptoms of COPD:

“I don't really know how to start asking questions. I don't know what I could do or what I could take to improve the situation. I didn't want to start trying different things like over the counter stuff to get pressure off my chest or get rid of the mucus. Also, I was scared that taking it would do more harm than good” (F2 P1 #1).

“I actually just found out that I had COPD. I wonder if the disease ever goes away? I don't know what I could do to help it or anything. Since I was just diagnosed, I am waiting on receiving information and then I can start to help treat myself. I see on TV that you are supposed to take longer walks and dance. I haven't tried any of those activities yet though” (F2 P2 #1).

Patients who had been living with COPD longer reported being better able to self-manage their disease; moreover, any lack of COPD self-management knowledge was related to the relative exposure that patients had dealing with COPD. Because of the variability within this sample in terms of years since diagnosis, this variation in COPD self-management knowledge was to be expected. The comments offered by the focus group seemed to support a continuum of patient knowledge, ranging from little to no knowledge (e.g., reflected by the comments above) to specific knowledge about ways to help manage disease-related symptoms. In particular, patients reported the regimented use of medication. All patients who had been dealing with COPD for a number of years reported feeling extremely confident in knowing how and when to use their prescribed medications:

“I think we all use medication and know how to use the medications. We feel well informed about how to use our medications, whether it be inhalers, nebulizers, or decongestant pills. We always have our pills and medications nearby” (F2 P1 #2).

“I can't sleep at night unless I have my mask on at night, so I have to know how to put that on” (F1 P3 #2).

In addition to medication management, patients noted the value of rest throughout the day and getting plenty of sleep at night. Apart from getting enough rest, however, patients reported a lack of knowledge and skills regarding self-management strategies which did not include taking their prescribed medication(s). Of considerable note was the absence of any patients practicing breathing exercises or controlled coughing, or using any specific relaxation techniques or energy conservation strategies. This was primarily because they had never been exposed to any seminars or informational resources which showed them how to perform these skills. It was interesting to note that patients were very inquisitive about types of activities or lifestyle changes that they could partake in to help reduce COPD symptoms. Patients seemed very interested when the conversation transitioned from focusing on medication management to focusing more on other new self-management strategies. Additionally, focus group participants raised certain questions and addressed specific learning needs related to COPD self-management. These comments included:

“Is there a certain type of food that you eat that has an effect on your breathing?” (F2 P1 #2).

“I want to learn more about exercise, because I'd like to walk more effectively” (F2 P2 #9).

“I want to learn more about relaxation, so I can stay more relaxed. I feel very comfortable saying so too” (F1 P6 #5).

“I want to be active. I want to do what I have to do to keep going” (F2 P1 #2).

Many patients remarked about how they had been encouraged to quit smoking but were unable to due to so. The reasons for being unable to quit ranged from cigarettes being too addictive to the excess expense of smoking cessation aids. Even though smoking cessation was regarded as extremely difficult, patients noted an expressed desire to “kick” the habit. This desire was motivated by patients associating with peers who had successfully stopped smoking.

#### 5.1.4. Coming to Terms with COPD and the Lifestyle

This subtheme reflected (a) how patients rationalize their COPD diagnosis and (b) how they describe the lifestyle prompted by COPD. Surprisingly, patients had various different beliefs regarding the origin of their affliction:

“I think that my diagnosis has something to do with being around fumes when I was working. It's either because of that or some of the stuff I used to do as a youngster” (F2 P1 #1).

“I never had problems until I moved down south, when I got chronic bronchitis” (F2 P2 #1).

“I didn't know why I had the disease. I never smoke, never drank. My ex-husband did, smoke, however. I didn't think I'd get the disease though, because we had been divorced since 1974” (F1 P6 #1).

Almost all of the patients in the focus groups acknowledged a need to listen to their doctor's advice regarding the self-care of COPD. Although some patients noted the initial tendency to resist doctors' orders when first diagnosed with COPD, most patients reported succumbing to the idea of a “disease-compliant” lifestyle. Patients began to realize that for life to be good, it would have to be different. Most focus group participants noted that they never regretted deciding to follow their doctor's advice.

While patients reported feeling annoyed by the deleterious impact that COPD had on their life satisfaction, they were cognizant that COPD is progressive and can get worse. Furthermore, they recognized through observing others that COPD can be extremely debilitating. Because of this, patients reported wanting to do everything they could to maintain their quality of life at a level superior to those whom they've observed to be far more affected by the disease:

“I know this [COPD] is not death though. It just works on your nerves and changes your whole lifestyle. Some people are a lot worse than me though. I just want every drop of information I can get about this thing” [COPD] (F2 P2 #1).

“It's really a day to day kind of disease. Now tomorrow I might try to do more, because it might be a better day tomorrow. I think that trying to do exercises might help expand my lungs, so I'll try to do more on days when I feel good” (F2 P1 #2).

### 5.2. Classical Content Analysis

To complement the results gleaned from the constant comparison analysis, a classical content analysis procedure was employed. After all of the data were coded (i.e., units of data were identified and classified into systematic categories that distinguished unique data properties), it was determined how many times each code was utilized. The most frequently broached concepts included: the inability of patients to carry out activities of daily living, a lack of knowledge regarding COPD self-management behavior modification strategies, and the differential learning curve for managing shortness of breath. Participants also frequently discussed the idea of not moving in excess for fear of breathlessness. Patients reported less frequently discussing the need for purposeful living, which revealed that their current lifestyles were not currently conducive to limiting dyspnea exacerbations.

**Figure 1:**
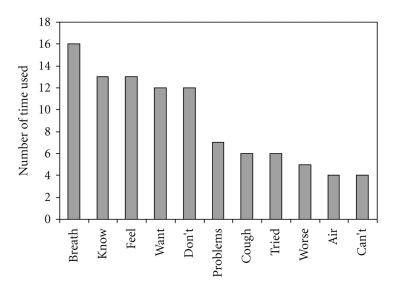
Most frequently used words during focus groups.

Figure 1 presents a chart that compares the frequency of the most commonly identified codes within the focus group data. While there existed some variability in the number of times each code was utilized, there was consensus regarding which subthemes (represented via codes) were most prevalent within the data. All of the frequently used codes were used to identify two of the subthemes identified in the present data. These commonly identified codes contributed to the makeup of two related subthemes: (a) *adjusting physically to COPD* and (b) *using COPD self-management to adjust physically to the disease*.

### 5.3. Word Count

**Figure 2:**
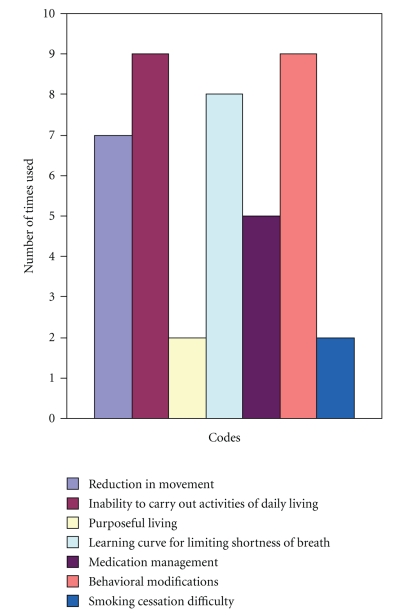
Results from classical content analysis.

Finally, a word count method was used to quantify the number of times a specific word was spoken during each of the focus group interviews. The word count was implemented on the final transcript using QSR International's NVivo 7.0 qualitative data analysis software program. This software enabled the seamless identification of word patterns present throughout the qualitative data [53]. The most commonly used words within the transcript were: *breath, know, feel, want, *and* don't*, with *breath* being the most commonly cited word. Patients overwhelmingly mentioned the word *breath* during the course of the focus groups, which revealed how important the maintenance of *breath* was to both their quality of life and overall perception of COPD. The participants had very strong *feeling*s related to *wanting* their shortness of *breath* to be limited. Patients were adamant in their desire to receive any *knowledge* that could help limit the onset of dyspnea exacerbations. Figure 2 depicts the frequency of the most commonly spoken words.

Lesser used, but frequently mentioned words such as *problems, tried, worse, *and* can't* were also of note. Focus group participants reported having *tried* to cope with the numerous *problems* caused by COPD; but, more often than not, patients reported their symptoms as becoming demonstrably *worse* no matter what they tried to do. What is more, patients could not readily identify multiple ways to limit the onset of shortness of breath. It was interesting to note the omnipresence of words such as *want *and* don't* as compared to *tried* and *can't*. The differential use of these specific words may provide unique insight into how patients view surviving and living life with COPD. While most all patients reported an expressed desire to *want* to do something to self-manage COPD, the efforts prompted by this desire did not (*don't*) necessarily result in the health outcomes they were looking for. Thus, the focus group participants seldom reported that they *tried* to actively limit their shortness of breath. Primarily, this was because patients *can't* successfully engage in activities (other than taking medications), which actually helped reduce their dyspnea. In light of this, most patients felt reduced to simply taking their medications, with little guidance regarding other options for COPD self-management.

## 6. DVD Development

RVision Corporation [55] developed a library of education and exercise rehabilitation content for home use by patients with COPD. These three COPD self-management educational segments cover almost 70 instructional topics. Topics include pursed lip and diaphragmatic breathing techniques, aerobics and conditioning, infection management/treatment, medication management, smoking cessation, aero chamber and bronchodilator use, energy conservation, relaxation techniques, cough controlling, nutrition, weight management, and walking for exercise. Previous work has shown that patient use of this specific content can result in improvements of quality of life, fatigue, and exercise compliance [38]. Table 3 lists content areas covered within each of the 3 segments. These segments run for approximately 1 hour, and 30 minutes in length in total. To condense these segments and target them to the learning needs of the participants in this study, video editing technology was used to compress the relevant patient education topics (identified during the qualitative data analysis) into a single interval of approximately 30 minutes. Each topical segment was placed in a unique chapter on each DVD. This was done to allow patients to specifically choose the segments they wanted to watch, without being forced to watch the entire DVD at each sitting. This was also done in recognition that there was going to be variability in the types of topics that patients might already be familiar with. Within each segment, sources of efficacy information included performance mastery techniques, role modeling, physiological coping, and verbal persuasion [56].

## 7. Discussion

In sum, the self-management experiences of COPD patients in these focus groups were limited to taking prescribed medications and reducing movement and activity. Patients reported widespread confidence with regards to managing and using prescribed medications to treat COPD symptoms, and they also perceived that getting plenty of rest could reduce their shortness of breath. However, the focus group participants did not realize the importance of practicing other COPD self-management skills and behaviors. By and large, patients reported a lack of knowledge and skill development related to alternative rehabilitative activities such as controlled breathing and coughing, stress reduction, smoking cessation, nutrition, and paced walking/activity. Furthermore, the focus group participants expressed an interest in learning more about these and other novel topics, which have universally been identified as staples within COPD self-management regimens [10]. It should be noted here that the social characteristics of this particular community may limit the generalizability of these particular educational needs. However, the findings from this study may generalize to communities which possess similar shared characteristics. In this primarily rural community, it can be surmised that needs identified from these focus groups can potentially be applied to other COPD patient populations represented by relatively equal proportions of African American and Caucasians who are living at or below regional poverty lines with a low socioeconomic status.

Given the learning needs identified earlier, the low educational levels of many of the patients in the sample, and the variability in terms of patient time spent coping with COPD, careful attention was paid to developing the DVD instructional tool based on patient feedback obtained during this exploratory study. It was imperative to only include segments that were comprehensible, to the point and clear. Segments which contained relatively novel COPD self-management education (determined based on input from patients) were selected for inclusion, while segments deemed irrelevant were not included. The edited DVD was not intended to bombard patients with an excess of information that they could not remember and use; rather, it was developed in hopes of keeping patients' interest by only including information that they would find to be applicable to their disease self-management efforts.

For example, there were no segments included on medication management, because most patients felt comfortable using their medications, and these segments tended to describe specific prescription medication terminology, which may have confused patients who did not explicitly remember the names of the medications that they were currently taking. Given that the majority of patients in the focus groups had been diagnosed for many years (Mean = 6 years; SD = 4.43 years), they professed being familiar with the medications they used regularly to management their disease. Had the focus groups or patient sample been composed of those more recently diagnosed with COPD, however, it would have been prudent to include information on prescription management and adherence to help assist patients in adapting to their new disease status. After taking into account input from the Medical Director of the clinic where the patients were treated, the DVD clips were edited and placed on an original disc. Table 4 presents the content areas included on the edited DVD and the running time for each segment.

The total run time for the entire edited DVD was approximately 34 minutes and 18 seconds. It was used in a study [57] to determine optimal self-management education strategies for COPD patients. Patients provided with a DVD reported watching the targeted segments multiple times (Mean = 2.58, SD = 1.81), perhaps because of the control they had over the implementation of the DVD player used to transmit the self-management education. Moreover, using DVD technology as the technological modality for this intervention gave COPD patients the greatest opportunity for empowerment over persistent shortness of breath which characterizes their disease. Patients could view the educational material in the convenience of their own home, using a technology they were familiar with, at their own discretion. Given the geographic and socioeconomic characteristics of this sample of COPD patients, the overarching instructional strategy supported limited interpersonal interaction with patients. Thus, a *distance education* method served as the primary means of instruction. For the purpose of this project, distance education can be defined best by Moore [58] as, “all arrangements for providing instruction through…electronic communications to people engaged in planned learning in a place or time different from that of the instructor or instructors” (p. xv).

In future dissemination studies using the targeted DVD within the distance learning milieu, various other telecommunications vehicles may be integrated into self-management education for rural patients with COPD. For example, the multimedia content reformatted for this project (i.e., to coincide with patient self-management education needs) has been posted on the internet by way of YouTube broadcasting using the feed managed by the proprietor of RVision Corporation: http://www.youtube.com/watch?v=pte_GGQb1_4. The authors posit that these types of interactive, media sharing URLs will allow COPD patients to contribute both video and text-based responses, comments and concerns as regards the self-management tutorials streamed over the internet. An added benefit of using the YouTube application is that patients subscribing to the feed will be afforded the opportunity to view related videos within the preexisting library of all health education segments produced by RVision Corporation. For the multitude of COPD patients that suffer from other, comorbid chronic conditions, the access to these libraries could prove to be an extremely valuable resource. RVision Corporation educational content for various diseases and disorders (including COPD) is commercially available and distributed by Health Ix via their website: http://www.healthix.com/. Each video clip posted for telemedical purposes could be dispensed using a unique host URL link.

This integration of video-based education in an open access web environment (e.g., YouTube) can provide a forum for patients to share their experiences attempting to cope with disease-related issues within a virtually networked community. For rural patients who have difficulty commuting to a common, localized site for health services and support, this portal containing instructional multimedia material is ideal. This converged approach to congregating patients and providers in a user environment amenable to patient/provider feedback can enable the site administrator to continually meet patient needs for self-management education. In addition, the administrator has the luxury of managing and optimizing a single network to transmit both audio-visual education and patient feedback asynchronously over the internet on a common system. This technologically-mediated strategy would reduce rural patients' need to travel and deliver the educational content directly to patients at their place of residence in the same vein as the DVD, but with the added networking capacity.

Access to this host URL could also be granted to certified medical personnel of rural health clinics to better assimilate the DVD content with tailored medical advice provided both through audio-visual and text-based responses. Special attention must be paid to ensuring that patient confidentiality is not breached when sharing medical information within a public domain forum on the internet. An encrypted web portal with VOIP software applications (e.g., Skype, Google Talk, Cisco IP Communicator, etc.) enabling two-way voice communication over the internet could assuage concerns regarding the unauthorized transmission of patient health information.

At this point, it should be noted that this future work will only become realized should pending financial stimulus be allocated to rural health networks for greater broadband access to the internet. Recent initiatives in the United States have suggested that this enhanced, internet access for rural America is forthcoming for these underserved areas. Furthermore, future researchers using this proposed converged networking approach to technologically-mediated disease self-management education should be confident that patients/providers are prepared with instruction and training to use the internet and all related applications. Without patient/provider access and efficacy with regard to internet/software navigation, the fruits of the internet as a telemedicine application can be very limited. It is suggested that such attempts be formatively pilot tested in areas of higher socioeconomic status, before broad-based implementation in rural, underserved areas is attempted. The aforementioned mobile COPD self-management education service, in a converged networking environment, would be especially useful in a technologically capable sample of rural COPD patients, because the majority of focus group participants expressed difficulty transporting themselves from their homes to their rural health clinic and often could only do so with assistance from friends and family members. Patient populations living in urban regions may not be affected by this geographic barrier; thus, the proposed intervention may be less appropriate in areas with a dense populous.

Focus group data proved extremely useful to identify and confirm patients' learning needs. Engaging in such formative qualitative inquiries may prove invaluable when attempting to meet the self-management education needs of diverse patients who are difficult to reach. It would stand to reason that results from this study suggest that the prospect of using self-management education DVD content could potentially stimulate high utilization rates among rural COPD patients, which could overcome significant barriers relative to widespread distribution of COPD self-management techniques. By disseminating targeted educational technology resources to underserved populations, health educators may be able to broaden the reach of COPD self-management messages and help patients feel more satisfied with the patient education they receive.

## Figures and Tables

**Table 1:** Focus group demographic characteristics.

Characteristics	*n* = 12
Age (mean ± SD)	67 years ± 8.07 years
*Gender *	
Male	4
Female	8
*Race*	
Caucasian	7
African American	5
*Educational level *	
Grade school	6
Some high school	2
High school graduate	2
Some college	0
College graduate	2
Graduate degree	0
*Income *	
Less than $14,999	0
$15,000–$24,999	12
$25,000–$34,999	0
$35,000–$49,999	0
$50,000–$74,999	0
$75,000–$99,999	0
$100,000+	0
*Marital Status*	
Married	6
Widowed	4
Single	2
*Living arrangements*	
With family	10
Alone	2
Number of years since diagnosis (mean ± SD)	6 years ± 4.43 years

**Table 2:** Theme and subthemes for data and associated codes.

Theme	Subthemes	Codes
Surviving and living well with COPD		(i) Reduction in movement
	- Adjusting physically to COPD	(ii) Inability to carry out activities of daily living
		(iii) Purposeful action
		(iv) Learning curve for limiting shortness of breath
	- COPD self-management:	(v) Medication management
	adjusting physically	(vi) Limited behavioral modifications
		(vii) Smoking cessation difficulty
		(viii) Beliefs about origin of disease
	- Coming to terms with COPD	(ix) Need for disease compliant lifestyle
	and the lifestyle	(x) Interest in attaining predisease quality of life
		(xi) Acknowledgement of progressive disease course

**Table 3:** Content covered within 3 RVision DVD segments.

Content	Running Time
*COPD Education Segment #1*	

Pursed Lip Breathing	55 seconds
Diaphragmatic Breathing	1 minute, 2 seconds
Energy Conservation	1 minute, 34 seconds
Introduction to Relaxation Techniques	35 seconds
Deep Breathing	22 seconds
Total Muscle Relaxation	1 minute, 39 seconds
Visual Imagery	21 seconds
Helpful Hints for Relaxation	24 seconds
Avoiding Stress	23 seconds
Panic Control Breathing	47 seconds
Bathing and Showering	1 minute, 8 seconds
Grooming	32 seconds
Dressing	44 seconds
Aerobics and Conditioning Introduction	23 seconds
Walking for Exercise	2 minutes, 37 seconds
Breath Saver Tips	37 seconds
Lifting and Breathing	15 seconds
Bending and Breathing	24 seconds
Going Up Stairs	39 seconds
Infection Control	1 minute, 41 seconds
Infection Detection	1 minute, 1 seconds
Infection Treatment	59 seconds
When to Call Doctor	1 minute, 17 seconds

*COPD Education Segment #2*	

Medication Introduction	30 seconds
Bronchodilators	36 seconds
Antibiotics	44 seconds
Metered Dose Inhalers	4 minutes, 10 seconds
Proper Use of Inhaler	41 seconds
Digoxin	33 seconds
Corticosteriods	42 seconds
Side Effects of Prednisone	1 minute, 5 seconds
Controlling your Cough	47 seconds
Nutrition	2 minutes, 39 seconds
Tips for Good Nutrition	6 minutes, 15 seconds
Increasing Your Fluid Intake	1 minute, 29 seconds
High Potassium Foods	1 minute, 2 seconds
Diet Hints	49 seconds
Weight Management	1 minute, 12 seconds
Eating Out	48 seconds
Avoid Constipation	34 seconds

*COPD Education Segment #3*	

Home Bicycle Program	1 minute, 58 seconds
House Keeping	1 minute, 11 seconds
Travel	1 minute, 55 seconds
Intimate Relations	2 minutes, 19 seconds
Smoking Cessation	1 minute, 22 seconds

**Table 4:** Final segments included on DVD (edited).

Content	Running Time
Introduction	49 seconds
Pursed Lip Breathing	55 seconds
Diaphragmatic Breathing	1 minute, 2 seconds
Energy Conservation	1 minute, 34 seconds
Introduction to Relaxation Techniques	35 seconds
Deep Breathing	22 seconds
Total Muscle Relaxation	1 minute, 39 seconds
Visual Imagery	21 seconds
Helpful Hints for Relaxation	24 seconds
Avoiding Stress	23 seconds
Panic Control Breathing	47 seconds
Aerobics and Conditioning Introduction	23 seconds
Walking for Exercise	2 minutes, 37 seconds
Lifting and Breathing	15 seconds
Bending and Breathing	24 seconds
Infection Control	1 minute, 41 seconds
Infection Detection	1 minute, 1 seconds
Infection Treatment	59 seconds
When to Call Doctor	1 minute, 17 seconds
Controlling your Cough	47 seconds
Nutrition	2 minutes, 39 seconds
Increasing Your Fluid Intake	1 minute, 29 seconds
House Keeping	1 minute, 11 seconds
Smoking Cessation	1 minute, 22 seconds
Conclusion	22 seconds

Total (approximate)	34 minutes, 18 seconds

## Appendices

### A. Focus Group Rubric

#### A.1. Introduction

Good morning/afternoon, and welcome to our session. Thank you for taking the time to join our discussion of COPD patient education needs and self-care. My name is Michael Stellefson, and I am a doctoral student at Texas A&M University in College Station, Texas, and I am studying patient education needs for COPD self-management. Today I'd like to hear from you about how you view caring for yourself and your COPD and what types of topics you would like to learn more about pertaining to how you can care for your disease. You were selected for this discussion because you all have a diagnosis of COPD and you are treated here at Choctaw Urgent Care. I am very interested in your views of your disease and your experiences trying to manage your breathing.

Today we basically want to learn from you about how you live with COPD and what types of disease self-management activities you'd like to learn more about. Please feel free to share your point of view whatever it may be, as there are no wrong answers to my questions. Please try to speak one at a time and speak up whenever you want to add something to the discussion. I am tape recording our conversations so that I don't miss any of your comments. We will be on a first name basis, and your names will not be attached to any of your comments to ensure your confidentiality.

My role here is to ask questions and listen. I will be asking about 10 total questions, and I'll move the discussion from one question to the next. As you talk and discuss issues, I will be taking notes about what you are sharing, so even if I'm writing, please continue with your comments, as I am trying to listen to everything you are saying. Please share as much as you can, but leave time for everyone to have the opportunity to share their thoughts and feelings. Also, please feel free to talk with one another. I've placed name cards on the table in front of you so that we can all remember each other's names during the discussion. We'll be done by (—), and I'll leave a little time at the end so you can finish the surveys in front of you. So to start…

#### A.2. Questions

1. Think back and tell us when you were diagnosed with COPD, and how long you have been dealing with health problems because of your disease?
2. What types of daily activities do you wish that you could do but don't feel able to do anymore because of having COPD?
3. When you think about trying to take care of yourself with your disease, what types of activities do you think about doing?
4. Of these activities, what do you do, yourself, do to help take care of your disease (i.e., things that you do so you don't feel short of breath or ill)? What allows you to do these activities?
5. What new and/or different activities did you maybe try to do to manage your disease but maybe did not feel comfortable continuing because of discomfort or difficulty? Can you think of any specific barriers that prevent you from trying to take care of yourself?
6. What questions do you have regarding what you can do to help limit the problems that you experience because of COPD?
7. Of all of these activities that we just discussed, what type of self-treatment activity do you consider most important?
8. What would like to learn more about in terms of taking care of yourself with your disease? What advice could you give someone who was trying to determine what to teach a patient with COPD?

### B. Member Check

1. How well does this summary capture what we discussed today? Does what I said sound accurate?
2. Is there anything that we did not talk about today regarding how you take care of your COPD that you would like to mention?
